# The measles virus matrix F50S mutation from a lethal case of subacute sclerosing panencephalitis promotes receptor-independent neuronal spread

**DOI:** 10.1128/jvi.01750-24

**Published:** 2024-12-06

**Authors:** Iris Yousaf, Luke Domanico, Toshihiko Nambara, Kalpana Yadav, Lauren K. Kelly, Jorge Trejo-Lopez, Wun-Ju Shieh, Paul A. Rota, Patricia Devaux, Takahisa Kanekiyo, Matthew P. Taylor, Roberto Cattaneo

**Affiliations:** 1 Mayo Clinic Graduate School of Biomedical Sciences, Mayo Clinic 32864 , Rochester, Minnesota, USA; 2 Department of Molecular Medicine, Mayo Clinic 4352 , Rochester, Minnesota, USA; 3 Department of Microbiology and Cell Biology, Montana State University 123776 , Bozeman, Montana, USA; 4 Department of Neuroscience, Mayo Clinic 633174 , Jacksonville, Florida, USA; 5 Department of Laboratory Medicine and Pathology, Mayo Clinic 195112 , Rochester, Minnesota, USA; 6 Infectious Diseases Pathology Branch, Division of High Consequence Pathogens and Pathology, Centers for Disease Control and Prevention 1242 , Atlanta, Georgia, USA; 7 Division of Viral Diseases, National Center for Immunization and Respiratory Diseases, Centers for Disease Control and Prevention 1242 , Atlanta, Georgia, USA; University Medical Center Freiburg, Freiburg, Germany

**Keywords:** measles virus, subacute sclerosing panencephalitis, brain disease, neurotropism, virus receptor, membrane fusion, population genetics, collective infectious unit, virus persistence, matrix protein

## Abstract

Subacute sclerosing panencephalitis (SSPE) is a lethal neurological disorder occurring several years after measles. Reconstruction of the evolution of the measles virus (MeV) genome in an SSPE case suggested that the matrix (M) protein mutation M-F50S, when added to other mutations, drove neuropathogenesis. However, whether and how M-F50S would promote spread independently from other mutations was in question. We investigated here the cell specificity of MeV spread in this brain and documented that both neurons and astrocytes were heavily infected. We then generated recombinant MeV with individual mutations in the three proteins of the viral membrane fusion apparatus, M, fusion (F), and hemagglutinin (H). These viruses reached similar titers as the parental wild-type virus, kept the respective mutations upon passage, and infected cells expressing the tissue-specific MeV receptors SLAM and nectin-4 with similar efficiencies. However, after inoculation of receptor-negative neurons and astrocytes differentiated from human induced pluripotent stem cells, only *MeV M-F50S* spread with moderate efficiency; the parental virus and its derivatives coding for a hyperfusogenic F protein, or for a cytoplasmic tail-mutated H protein, did not spread. When delivered to primary mouse neurons by cell-mediated neurite overlay, *MeV M-F50S* frequently reached the cell bodies and occasionally formed small infectious centers, while the other MeV reached the cell bodies only sporadically. These results demonstrate that, in neuronal cell cultures, M-F50S can enable receptor-independent spread in the absence of other mutations, and validate the inference that this single amino acid change initiated ubiquitous MeV brain spread.

**IMPORTANCE:**

Measles virus (MeV), a non-integrating negative-strand RNA virus, rarely causes subacute sclerosing panencephalitis (SSPE) several years after acute infection. During brain adaptation, the MeV genome acquires multiple mutations reducing the dependence of its membrane fusion apparatus (MFA) from an activating receptor. It was proposed that one of these mutations, matrix protein F50S, drove neuropathogenesis in an SSPE case. We report here that, in two types of neuronal cultures, a recombinant MeV with only this mutation gained receptor-independent spread, whereas viruses expressing MFA proteins with other mutations acquired during brain adaptation did not. Our results validate the inference that M-F50S initiated ubiquitous MeV brain spread resulting in lethal disease. They also prompt studies of the impact of analogous amino acid changes of the M proteins of other nonsegmented negative-strand RNA viruses on their interactions with membrane lipids and cytoskeletal components.

## INTRODUCTION

Measles remains a leading cause of death among children because it suppresses immune function, and it is extremely contagious (1–3). Although measles is easily preventable, a rise in hesitancy to vaccination and 61 million vaccine doses postponed or missed during COVID-19 are causing larger outbreaks worldwide (4–7).

Measles virus (MeV) takes advantage of two cellular proteins expressed tissue specifically to spread sequentially in selected cells and organs of the human body: the signaling lymphocytic activation molecule (SLAM) (8) mediates MeV entry in alveolar macrophages and dendritic cells that deliver the infection to local lymph nodes (9, 10). After infection amplification in lymph nodes and primary lymphatic organs, multitudes of infected circulating immune cells deliver cell-associated virus to upper airway epithelia cells that express nectin-4 (11–15). Cell-associated multi-genome transmission contributes to rapid MeV adaptation to lymphocytic and epithelial tissues (16–18).

One of about 10,000 individuals infected by a wild-type MeV strain succumbs to subacute sclerosing panencephalitis (SSPE) 3–20 years after acute infection (19–21). Cell-free MeV particles cannot be isolated from brain autopsy materials of SSPE cases, but the virus spreads cell-to-cell (22). MeV replicating in the brain of SSPE patients acquire mutations that lower the activation energy threshold of their membrane fusion apparatus (MFA) and promote receptor-independent cell-cell fusion (23–33). The MeV MFA is constituted by three proteins, the particle assembly organizer matrix (M) protein, the fusion (F) protein, and the receptor-attachment protein hemagglutinin (H). In different SSPE cases, mutations either impair the expression and particle assembly organizing function of the M protein (23–27) or reduce the F protein stability and favor its receptor-independent activation, or truncate the F protein cytoplasmic tail, uncoupling the fusion inhibitory function exerted by the M protein (28–33).

Since animal models cannot adequately replicate the selective environment of the human brain, most insights in adaptation mechanisms favoring MeV persistence were derived from analyses of human brain autopsy materials (19, 34, 35). These studies stalled after the widespread adoption of the measles vaccine, which eliminated SSPE in countries with sufficient immunization (36). Thankfully, the entire frozen brain of an SSPE victim was donated, and we collected specimens from 15 locations (37). Deep sequencing analyses revealed that MeV RNA reads accounted for up to 20% of the ribosome-depleted RNA reads in forebrain specimens. These analyses covered the MeV genome in average 0.89 million times per base, while previous studies were limited to single digits genome coverage (38–40).

The deep sequencing analyses provided important new insights in the mechanisms of MeV spread in the brain. Surprisingly, two major MeV genome subpopulations were present at variable frequencies in all brain specimens. Most infected cells carried both genome types, named G1 and G2, suggesting the possibility of genetic complementation. Both G1 and G2 accumulated many mutations, including some proposed to be drivers of neurotropism acquisition similar or identical to mutations promoting receptor-independent spread in other SSPE cases (23–33).

Clonal deconvolution analyses reconstructed the evolution of the MeV genome population that spread in this brain; this population has attributes of a collective infectious unit (CIU) (41–45). It was inferred that an ancestral MeV genome may have entered the brain frontal cortex, and that during replication in this location, three potential driver mutations were selected: M-W125*, F-L454M, and F-Q527*. It is thought that further diversification of the ancestral MeV genome in the brain emergence site produced G1 and G2, and that acquisition of the M-F50S mutation by G1 may have enabled its co-migration with G2 out of the frontal cortex. Finally, frequency modulation of the glycoprotein cytoplasmic tail mutations F-Q527* and H-I8T by loss or gain on either G1 or G2 may have supported slow but steady brain spread by adjusting membrane fusion efficiency (37).

Since these evolutionary analyses suggested that, in this SSPE case, M-F50S mobilized the CIU and started its lethal brain spread, we sought to assess whether this mutation alone promotes receptor-independent neuronal spread. To test this hypothesis, we generated a recombinant MeV coding only for M-F50S. As controls, we also generated a MeV expressing an F protein with reduced ectodomain stability, F-L454M, and another expressing a cytoplasmic tail-mutated H protein, H-I8T. All recombinant viruses coded for a fluorescent reporter protein allowing direct identification of infected cells. We report that only the virus coding solely for M-F50S, named *MeV M-F50S*, acquired receptor-independent spread capacity in two systems: neurons differentiated from human-induced pluripotent stem cells (hiPSC) and primary mouse neurons.

## RESULTS

### Pathogenic consequences of MeV replication

We analyzed the pathogenic consequences of MeV replication in the brain of a 24-year-old patient who succumbed to SSPE 14 months after the onset of clinical symptoms (37). At autopsy, temporal lobe tissue was obtained, formalin fixed, and paraffin embedded.

**Fig 1:**
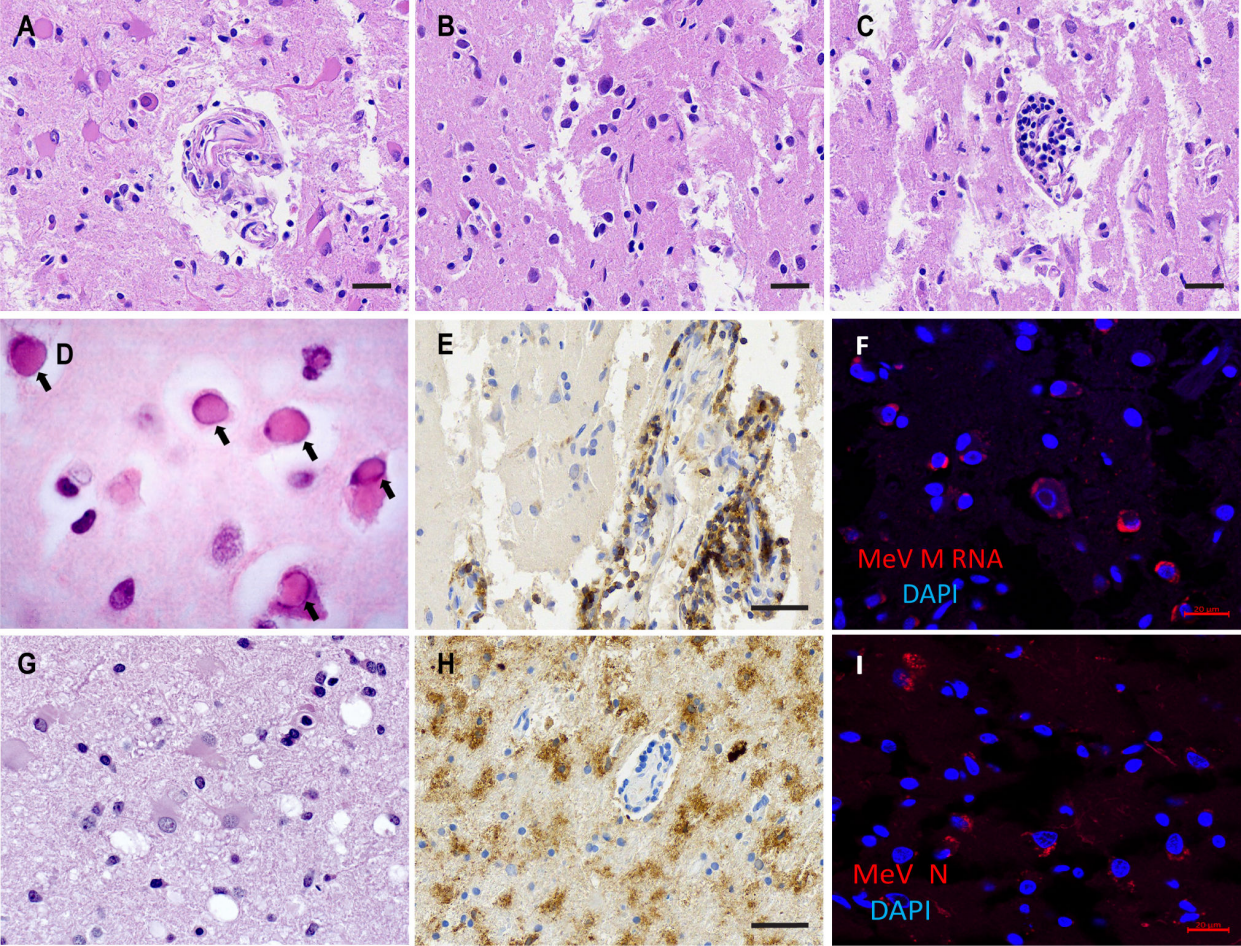
Histopathology of SSPE brain tissues. (**A, B, C, D**) Hematoxylin and eosin staining of temporal lobe tissue documenting (**A**) reactive gliosis; (**B**) mixed chronic inflammation; (**C**) perivascular chronic inflammation; scale bars for A through C: 30 µm. (**D**) Cowdry type-A inclusion bodies (arrows). (**E**) CD45 staining for hematopoietic cells. Scale bar: 50 µm. (**F**) MeV M mRNA detection by *in situ* hybridization (red). Nuclei are counterstained with DAPI (blue). Scale bar: 20 µm. (**G**) Hematoxylin and eosin staining of uninfected brain control tissue. (**H**) CD11c staining for dendritic cells. Scale bar: 50 µm. (**I**) Immunofluorescence analysis of MeV N protein (red) localization in temporal lobe tissue. Nuclei are counterstained with DAPI. Scale bar: 20 µm.

Fig. 1A through D shows hematoxylin and eosin staining: reactive gliosis (A), mixed chronic inflammation (B), perivascular lymphoplasmacytic infiltrates (C), and Cowdry type-A inclusion bodies were identified in neurons (D), consistent with viral infection. Fig. 1E documents large perivascular lymphocytic infiltrates that are CD45+, and Fig. 1H identifies numerous CD11c+ activated microglia within the parenchyma. Similar observations were done in specimens from the occipital lobe and another part of the forebrain. Fig. 1G shows hematoxylin and eosin staining of an uninfected control brain tissue slice.

We also assessed MeV transcription and translation in the temporal lobe, occipital lobe, and forebrain specimens. To detect MeV mRNA, we used single-molecule fluorescence *in situ* hybridization (smFISH) of M gene transcripts. Fig. 1F shows concentrated perinuclear and some diffuse cytoplasmic signals for MeV mRNA in many cells (red color, nuclei are counterstained blue with DAPI). To detect MeV protein, we used nucleocapsid (N) protein immunofluorescence analyses. Fig. 1I shows that N protein (red color, nuclei are counterstained blue with DAPI) was often concentrated perinuclearly possibly in MeV replication centers (46).

We used ImageJ to count MeV mRNA-positive (mRNA+) or protein positive (protein+) cells from a total of about 200 cells in the three brain specimens. In the temporal lobe, we detected 17.7% mRNA+ and 12.2% protein+ cells. In the occipital lobe, 9.1% of the cells were RNA+ and 6.8% protein+. And in the forebrain specimen, 21.0% cells were RNA+ and 19.4% protein+. Thus, MeV actively replicated in a significant fraction of the cells in all brain specimens examined.

### Both neurons and astrocytes are infected

**Fig 2:**
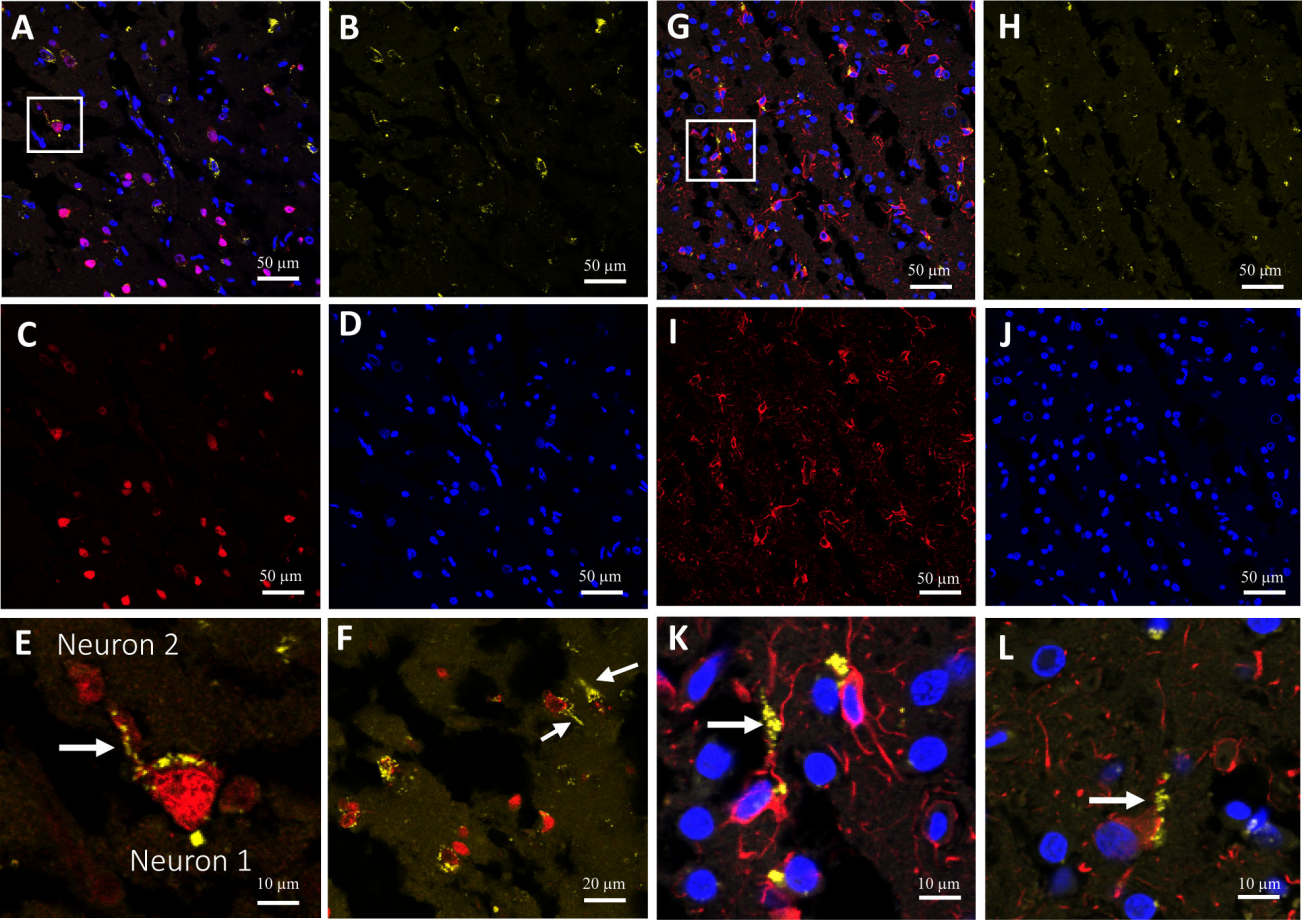
MeV replicates in neurons and astrocytes in SSPE brain tissue. (**A–F**) Immunofluorescence staining of a temporal lobe specimen showing merge (**A**) and individual channels for MeV nucleocapsid (N) protein (B, yellow), the NeuN neuronal marker (C, red), and DAPI (D, blue). (**E**) Zoomed in inset from panel A showing MeV N in axonal neurites (white arrow). (**F**) Image from occipital lobe specimen showing MeV N in axonal neurites (white arrows). (**G–L**) Immunofluorescence staining of temporal lobe specimen showing merge (**G**) and individual channels for MeV N (H, yellow), the GFAP astrocyte marker (I, red), and DAPI (J, blue). (**K**) Zoomed in inset from G showing MeV N (yellow) present at the distal ends of astrocyte (white arrow). (**L**) Image from cerebrum showing MeV N in distal ends of astrocyte (white arrows).

Previous reports documented MeV antigens predominantly in neurons (39, 47, 48) and also in oligodendrocytes and astrocytes (48, 49). To identify the cell types infected in this brain, we co-stained temporal lobe tissue sections for MeV N protein and the neuronal marker NeuN or the astrocyte marker GFAP. Fig. 2A through D and G through J show that both neurons and astrocytes, respectively, were infected with similar efficiencies. Notably, infected cells were often located adjacent to non-infected cells, and infectious centers or syncytia were not detected. While most MeV N signal was perinuclear, in some infected neurons, N signal was detected along the axonal neurites (Fig. 2E and F, white arrows). Distal MeV N signal was also observed in astrocytes (Fig. 2K and L, white arrows).

We then used ImageJ to quantitatively examine heavily infected areas. In a neuron-rich area of the occipital lobe, 50.4% of neurons were infected. In an astrocyte-rich area of the forebrain, 60.2% of astrocytes were infected. And in an area of the temporal lobe rich in both cell types, 40.2% of neurons and 63.3% of astrocytes were infected. Since the images used for quantification were taken in heavily infected areas, the percentages of infected neurons and astrocytes are not representative. Nevertheless, in this brain both neurons and astrocytes were efficiently infected.

### Recombinant MeV with individual mutations are genetically stable

Evolutionary analyses suggested that, in this SSPE case, M-F50S, when added to other mutations, drove neuropathogenesis (37). However, whether and how M-F50S would promote spread independently from other mutations was in question. Here, we sought to assess whether this mutation alone promotes receptor-independent neuronal spread.

**Fig 3:**
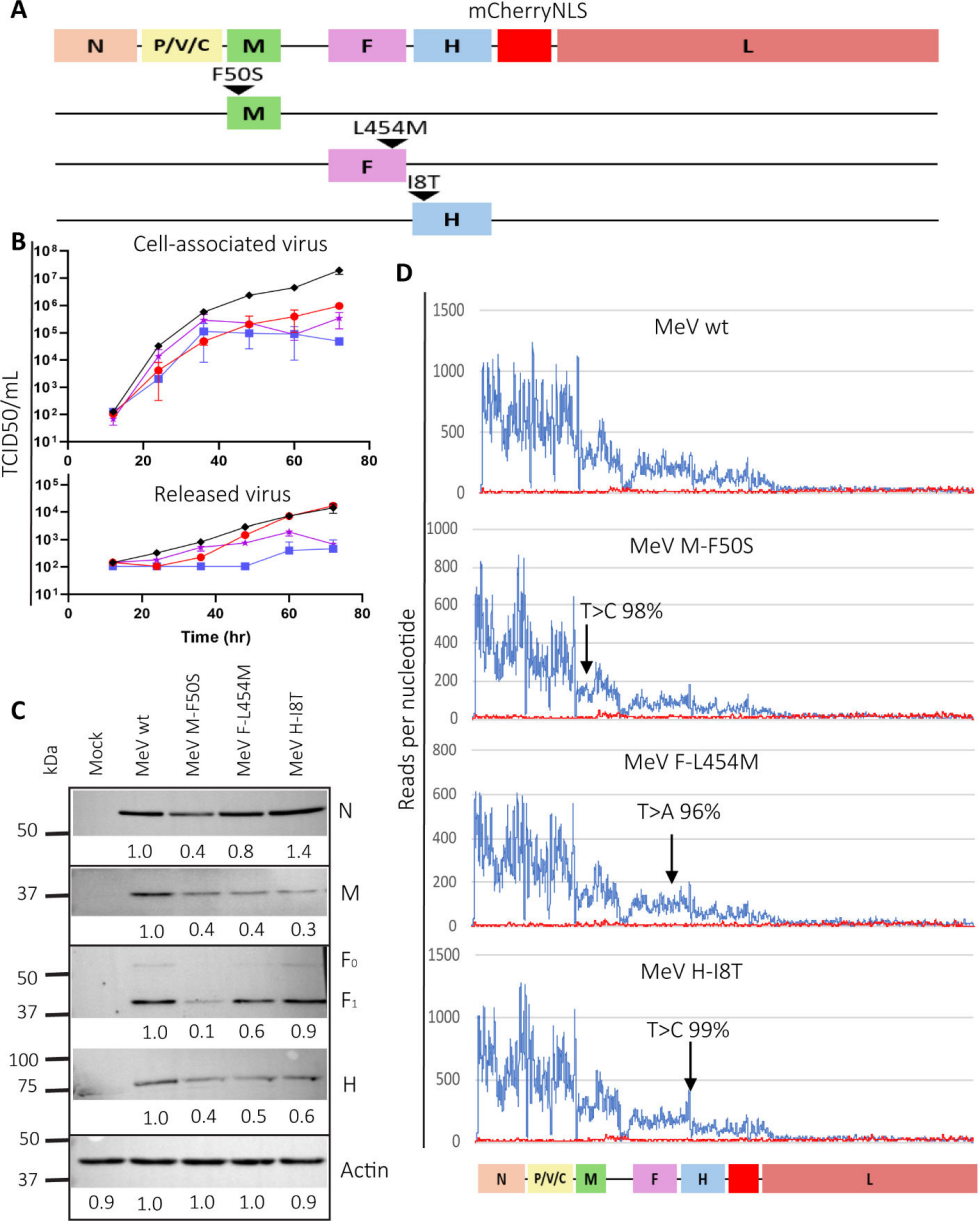
Characterization of recombinant MeV. (**A**) Schematic of the genomes of recombinant MeV with individual candidate brain tropism acquisition driver mutations. The coding regions of the six MeV genes are indicated with colored rectangles. Genomes include the coding region of a mCherry reporter protein with a nuclear localization signal (mCherryNLS) that concentrates the fluorescent signal in the nucleus. The name and approximate position of the three mutations are indicated above the reading frames of the respective genes. (**B**) Growth kinetics for the parental virus and its three derivatives in HeLa hSLAM cells infected at an MOI of 0.05. Cell associated (top) and supernatants (bottom) fractions were collected every 12 h and titrated on Vero hSLAM cells. Black indicates *MeV wt* titers (TCID50/mL), red indicates *MeV M-F50S* titers, blue indicates *MeV F-L454M* titers, and purple indicates *MeV H-I8T* titers. Two biological replicates with two technical replicates are shown. (**C**) Western blots detecting the expression of the MeV proteins N, M, F, and H, and of cellular beta-actin. Intensities of the signals are indicated below each band as relative values compared to the corresponding MeV wt signal intensity. HeLa-hSLAM cells were infected at an MOI of 0.3 and harvested at 24 hpi. (**D**) Deep sequencing analyses of parental and mutant MeV stocks documenting the stability of the mutations introduced. The percentage of reads corresponding to the introduced mutations is indicated. *x*-axis: MeV genome. *y*-axis: number of reads per nucleotide. Blue lines represent positive strand reads (MeV mRNA) and red lines represent negative strand reads (MeV genomic RNA). Part of the GC-rich region between the M and F open reading frames was inefficiently sequenced for technical reasons.

To test this hypothesis, we generated a recombinant MeV differing from the parental wild-type strain in a single amino acid, M-F50S (Fig. 3A, top). As controls, we generated MeV differing from the parental strain in single F or H protein amino acids, F-L454M or H-I8T, respectively (Fig. 3A, middle and bottom). F-L454M is like F-L454W, a mutation previously shown to destabilize the ectodomain of the F protein and to enhance neuropathogenesis (50). H-I8T alters the cytoplasmic tail of the H protein that interacts with M to stabilize the MFA (32). We introduced these mutations in a MeV wild-type genomic backbone coding for the mCherry fluorescent protein with nuclear localization signal (16). All recombinant viruses, *MeV F-L454M*, *MeV M-F50S*, and *MeV H-I8T,* were rescued without the need for parental protein complementation.

To characterize the infections of these viruses, we inoculated HeLa-hSLAM cells at multiplicity of infection (MOI) of 0.05. Fig. 3B shows kinetic analyses of titers measured intracellularly and in the supernatant. Parental *MeV wt* reached the highest cell-associated and released titers (Fig. 3B, black symbols and lines). The mutant viruses had slightly slower kinetics and reached lower titers. The *MeV M-F50S* growth kinetics was closest to that of the parental virus (Fig. 3B, red lines). *MeV F-L454M* had the lowest supernatant titers, consistent with the hyperfusogenic and cell-associated nature of a MeV with the L454W mutation (31, 51) (Fig. 3B, blue lines). Fig. 3C shows the protein expression levels of the parental and recombinant viruses in HeLa-hSLAM-infected cells. All the mutated viruses expressed slightly reduced levels of the M and H proteins compared to the parental virus, and F protein expression was lower in cells infected with *MeV M-F50S*.

To assess the genomic stability of the recombinant MeV, we deep-sequenced total RNA from cells infected with our virus stocks. Fig. 3D shows analyses of polarity and distribution of the sequencing reads. For all viruses, positive strand reads (blue lines) decreased progressively with the distance of the six genes from the 3′ end of the genome, reflecting transcriptional attenuation at gene junctions, as expected (52). For all viruses, negative strand reads (red lines) were detected at a constant level, indicating the absence of small defective genomes (46).

Whole-genome nucleotide variance analyses documented that the three mutations introduced were maintained in a large majority of the genomes (>99% in *MeV H-I8T*, >98% in *MeV M-F50S,* and >96% in *MeV F-L454M*). The whole-genome analyses revealed variability of a few positions in different virus stocks, but variable nucleotides did not exceed 20% in any coding region position (Materials and Methods). Thus, all recombinant MeV generated here reach adequate titers and have operational genetic stability.

### Only *MeV M-F50S* spreads in hiPSC-derived neurons and astrocytes

**Fig 4:**
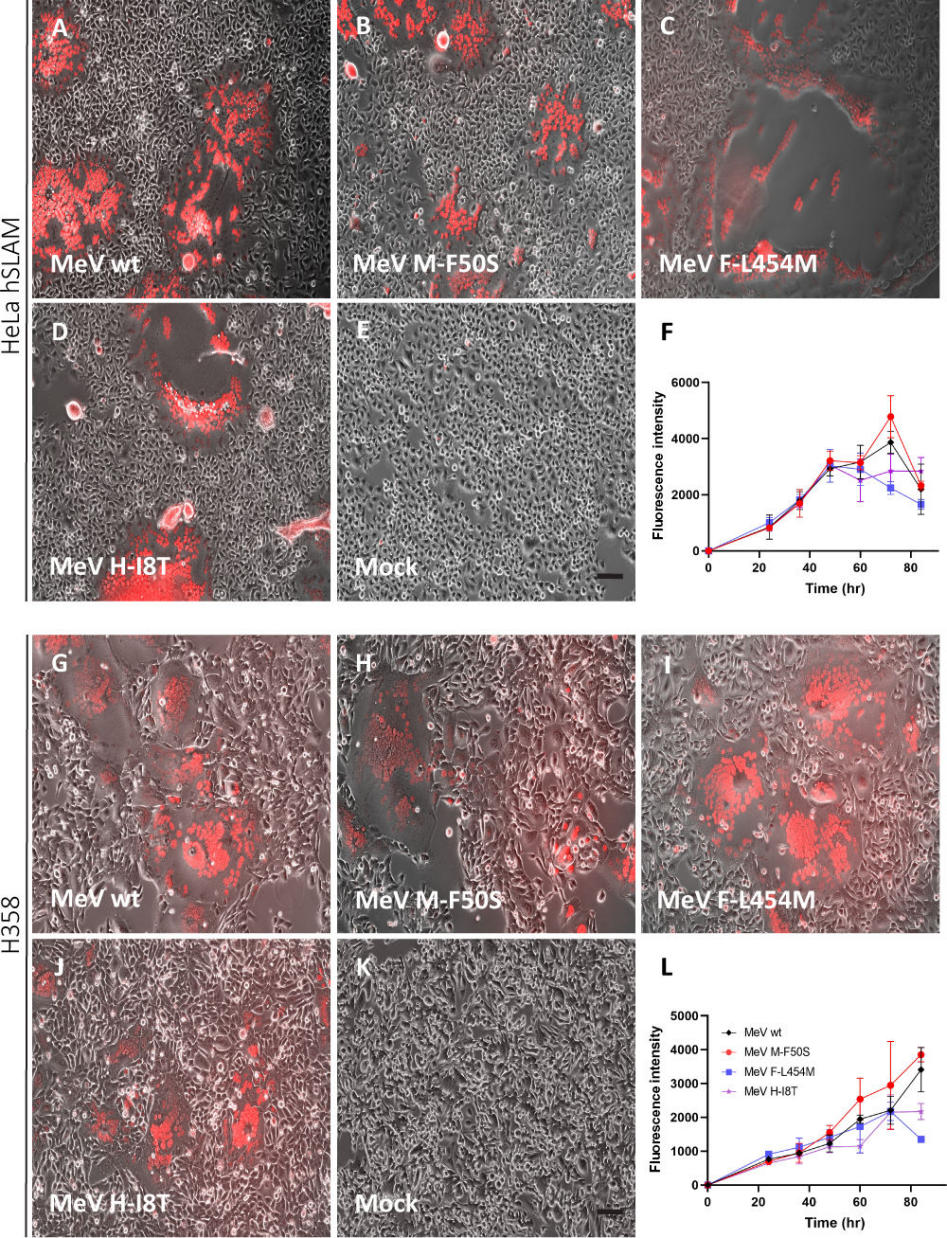
Robust infection of receptor-positive cell lines by all recombinant MeV. (**A–E**) Phase contrast images overlaid with fluorescence microscopy images of HeLa-hSLAM cells infected with the viruses indicated in the bottom left corners. Cells were infected at MOI 0.05, and images were taken 48 hpi. Scale bars in panels E and K: 100 µm. All photographs were taken at the same magnification. (**F**) Kinetic analysis of the fluorescence intensity (vertical axis) over time (hr, horizontal axis). Black indicates *MeV wt* intensities, red indicates *MeV M-F50S*, blue indicates *MeV F-L454M,* and purple indicates *MeV H-I8T*. *n* = 3. Fluorescence intensity was calculated by ImageJ. The low value for the *MeV F-L454M* infection at 84 hpi is due to extensive cell lysis. (**G–K**) Phase contrast images overlaid with fluorescence microscopy images of H358 cells infected with the viruses indicated in the bottom left corners. Cells were infected at MOI 0.05 and images were taken 72 hpi. (**L**) Kinetic analysis of the fluorescence intensity (vertical axis) over time (hr, horizontal axis). Black indicates *MeV wt* intensities, red indicates *MeV M-F50S*, blue indicates *MeV F-L454M,* and purple indicates *MeV H-I8T*. *n* = 3. Fluorescence intensity was calculated by ImageJ.

**Fig 5:**
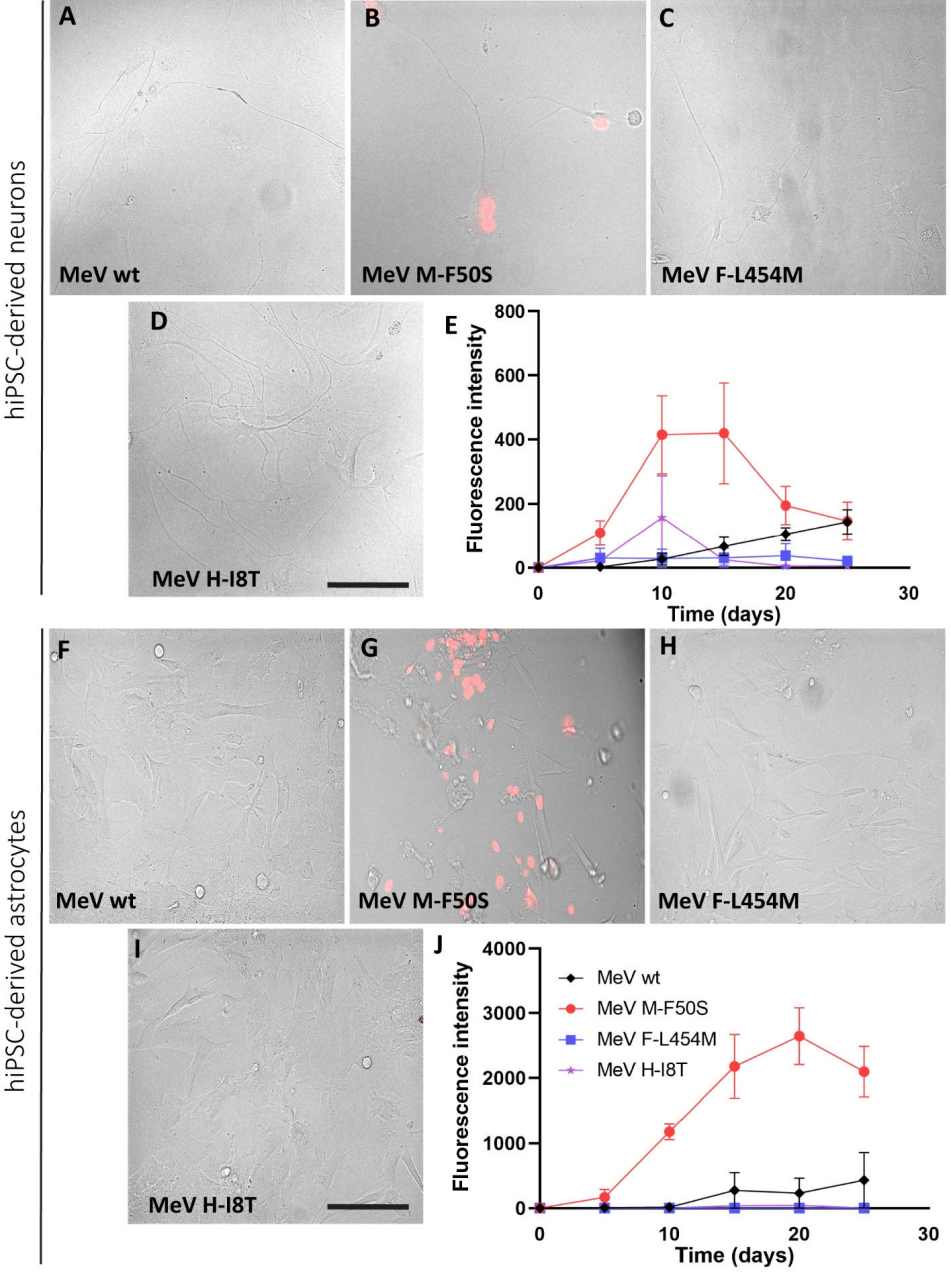
*MeV M-F50S* infects hiPSC-derived neurons and astrocytes more efficiently than the other recombinant viruses. (**A–D**) Phase contrast images overlaid with fluorescence microscopy images of hiPSC-derived neurons infected with the viruses indicated in the bottom left corners. Cells were infected at MOI 0.5, and images were taken 15 days post-infection. Scale bars in panels D and I: 200 µm. All images were taken at the same magnification. (**E**) Kinetic analysis of the fluorescence intensity (vertical axis) over time (days, horizontal axis). Black indicates *MeV wt* intensities, red indicates *MeV M-F50S*, blue indicates *MeV F-L454M,* and purple indicates *MeV H-I8T*. *n* = 3. Fluorescence intensity was calculated by ImageJ. (**F–I**) Phase contrast images overlaid with fluorescence microscopy images of hiPSC-derived astrocytes infected with the viruses indicated in the bottom left corners. Cells were infected at MOI 0.5, and images were taken 15 days post-infection. (**J**) Kinetic analysis of the fluorescence intensity (vertical axis) over time (hr, horizontal axis). Black indicates *MeV wt* intensities, red indicates *MeV M-F50S*, blue indicates *MeV F-L454M,* and purple indicates *MeV H-I8T*. Fluorescent intensity was calculated by ImageJ.

We then compared infectivity and fusogenicity of three MeV with individual mutations with those of the parental virus in cell lines expressing or not a MeV receptor. Fig. 4A through F shows analyses in SLAM-expressing cells and Fig. 4G through L in nectin-4 expressing cells; Fig. 5A through E shows analyses in receptor-negative hiPSC-derived neurons and Fig. 5F through J in receptor-negative hiPSC-derived astrocytes. Expecting slower infections in receptor-negative cells (50), we infected these cells at a 10-fold higher MOI than the receptor-positive cells (0.5 vs 0.05 MOI).

Fig. 4 documents infections of receptor-positive cells. These cells were robustly infected with all viruses, and all viruses induced the formation of large syncytia. In HeLa-hSLAM cells, syncytia formation started around 24 hours post infection (hpi) (Fig. 4A through E shows merged fluorescent and phase contrast images taken at 48 hpi). In nectin-4 expressing H358 cells, syncytia formation started later and was less extensive (Fig. 4G through K shows images taken at 72 hpi) consistent with previous studies (13). Syncytia appeared larger and cell death was faster in cells infected with *MeV F-L454M* than in cells infected with the other viruses (Fig. 4C and I). Fast spread through cell-cell fusion may account for earlier but lower peaks of infections for *MeV F-L454M* (Fig. 4F and L).

Fig. 5 documents infections of receptor-negative hiPSC-derived neurons and astrocytes. *MeV M-F50S* infected both cell types more efficiently than the parental *MeV wt* and the other mutant viruses (Fig. 5A through D and F through I). ImageJ analysis documented higher expression levels of the fluorescent reporter protein in hiPSC-derived astrocytes (Fig. 5J) than in neurons (Fig. 5E), but the density of astrocytes in this experiment was higher than that of neurons. These data indicate that only *MeV M-F50S* acquired receptor-independent spread.

### Only *MeV M-F50S* spreads efficiently in primary mouse neurons

To increase the efficiency of MeV delivery to receptor-negative cells, we took advantage of nectin-elicited cytoplasm transfer (NECT), a process transferring cytoplasmic materials, including MeV encapsidated genomes (ribonucleocapsids, RNP), from infected nectin-4 expressing cells to nectin-1 expressing neurons (53). To quantitatively assess the transfer of MeV RNP to neurons, we adapted the Campenot chamber system (54).

**Fig 6:**
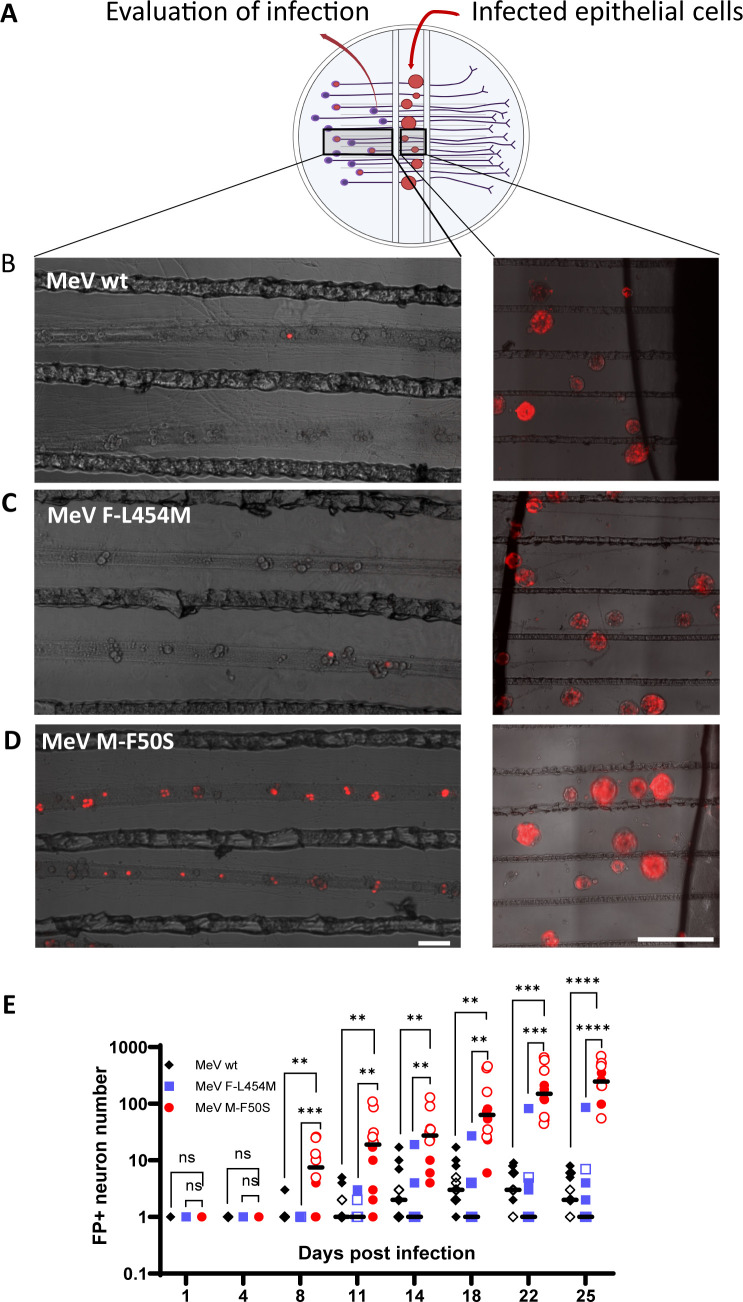
*MeV M-F50S* infects primary SCG neurons more efficiently than *MeV wt* and *MeV F-L454M*. (**A**) Schematic of the nectin-elicited cytoplasm transfer-based methodology used to deliver recombinant MeV infections to the compartmentalized primary mouse SCG neuron culturing system. (**B–D**) LEFT PANELS. Epifluorescence microscopy images acquired 14 days post-infection of SCG neurons in the soma compartment. Scale bars: 100 µm. RIGHT PANELS. Epifluorescence microscopy images of H358 syncytia infected with (**B**) *MeV wt,* (**C**) *MeV-F-L454M,* and (**D**) *MeV-M-F50S* in the neurite compartment. Scale bars: 500 µm. Images were acquired with equivalent laser intensity and exposure time; the 10× phase/TRITC channel merge was optimized for visualization of mCherry detection. (**E**) Kinetic analyses of SCG cell bodies infections. Neurites in the middle chamber were overlaid with 2 x 10^4^ MeV-infected H358 cells inoculated at an equivalent MOI of 0.5. Statistical comparisons were performed via one-way ordinary ANOVA using Tukey’s multiple comparisons test with a single pooled variance for each time point for two biological replicates. Data points from one biological replicate are shown with solid shapes and for the second with hollow shapes. Data points with a zero value are not shown. Median values other than zero are indicated with a black trait. Black shapes indicate *MeV wt*, red indicates *MeV M-F50S,* and blue indicates *MeV F-L454M*. *n* = 2 quintuplicate infection experiments. *P* values: * ≤0.05, ** ≤0.01, *** ≤0.00, **** ≤0.0001.

In this system, superior cervical ganglia (SCG) neurons are cultured within a Teflon ring. The ring is divided into compartments and attached to a treated culture surface, creating three hydrodynamically isolated compartments (Fig. 6A). Dissociated SCG neurons are plated in the left compartment and extend neurites underneath the internal walls of the ring. The isolated neurites in the middle compartment are then overlaid with MeV-infected H358 cells (Fig. 6A, middle compartment). Infection of neuronal cell bodies requires the transfer of MeV RNP into the isolated neurites followed by transport to the cell body (Fig. 6A, left compartment). As previously shown, a single barrier between neurite termini and soma can be used to experimentally evaluate transport of infection (55).

We overlaid H358 epithelial cells infected either with parental *MeV wt* or with *MeV F-L454M* or with *MeV M-F50S*, respectively, on neurites growing into the central compartment of the Campenot chamber system (Fig. 6B through D, right panels). Following retrograde-directed spread, MeV infections were detected in the cell bodies of the SCG neurons seeded into the left-most compartment (Fig. 6B through D, left panels; the signals detected correspond to the nuclei of infected cells, where mCherry accumulates).

Fig. 6E shows the numbers of fluorescence-positive SCG neurons observed over time in two quintuplicate infection experiments. Infections developed slowly, as in hiPSC-derived neurons. *MeV M-F50S* infections were most efficient: 14 days post infection (dpi) > 10 infected cell bodies were detected in most chambers, and 25 dpi all but one chamber had >100 infected cell bodies. *MeV F-L454M* and *MeV wt* had 1 or 2 chambers with >10 infected cell bodies 14 dpi and 0 (*MeV wt*) or 1 (*MeV F-L454M*) chambers with >100 infected cell bodies 25 dpi. In *MeV M-F50S* inoculated chambers, infected soma was detected more distally to the middle compartment than in chambers inoculated with the other two viruses. Visual assessment of infections revealed that *MeV M-F50S* inoculated cultures had small clusters of infected cells (Fig. 6D, left panel). Thus, only *MeV M-F50S* acquired preferential spread in primary mouse neurons.

To assess whether mutations were selected during *MeV M-F50S* replication in SCG neurons, RNA was extracted and deep sequenced. The F-I259T allele, present at <20% frequency in the virus amplified on Vero-hSLAM, was found at 41% frequency after replication in SCG neurons. This allele may have been positively selected, but we do not know whether this event is reproducible. No additional position at >20% variance was detected in the parts of the genome covered by >100 reads. Thus, *MeV M-F50S* spreads in primary mouse neurons without strong selection for additional mutations.

## DISCUSSION

A rise in vaccination hesitancy and millions of vaccine doses missed during the COVID-19 pandemics are causing larger measles outbreaks worldwide and foreshadow an SSPE comeback. To gain better understanding of this disease, we characterized the pathogenic consequences of MeV replication in the brain of a 24-year-old patient who succumbed to SSPE 14 months after the onset of clinical symptoms. We documented SSPE hallmarks such as perivascular lymphoplasmacytic infiltrates, reactive gliosis, microglial activation, and Cowdry type-A inclusion bodies in neurons (40, 47, 56). Extensive immune infiltration was observed as well (57, 58). Viral replication was robust, but no syncytia or infectious centers were detected, consistent with cell-to-cell viral spread, potentially occurring at synapses (22).

Previous analyses of SSPE cases documented MeV antigens predominantly in neurons (48, 59) but also at lower levels in oligodendrocytes and astrocytes (48, 49). In the SSPE brain examined here, infection of both neurons and astrocytes was robust. In these cell types, most signals were perinuclear, as shown previously for MeV-infected cultured cells (46). We detected MeV N protein aggregates in axonal neurites and in protrusions connecting adjacent neurons (Fig. 2E and F). MeV N protein was also detected near the distal ends of astrocyte protrusions (Fig. 2K and L). Movement of encapsidated genomes along neuronal axons has been documented for neurotropic viruses (60, 61); our findings suggest that encapsidated MeV genomes may also move along neuronal axons. It is unclear whether MeV infections can be transmitted between neurons and astrocytes *in vivo*, but noncanonical MeV transmission between these two cell types was recently documented *in vitro* (62).

How MeV reaches the brain is unclear. Another member of the morbillivirus genus, canine distemper virus, can reach this organ through two pathways: anterogradely via the olfactory nerve and hematogenously through the choroid plexus and cerebral blood vessels (63). MeV may enter the human brain through the olfactory nerve: infected nectin-4-expressing airway epithelial cells can transfer MeV to nectin-1-expressing neurons (53). Consistent with entry through the olfactory nerve, in this SSPE case, the frontal cortex was identified as the site of initial MeV genome amplification (37).

Our understanding of how MeV spreads in the brain is evolving. Human neurons do not express the MeV receptors SLAM or nectin-4, and the wild-type F protein cannot support membrane fusion (64). However, hyperfusogenic F protein mutants from SSPE patients can support membrane fusion in neurons (31, 33, 65). This process relies on the availability of brain-specific isoforms of two cell adhesion molecules, CADM1 and CADM2, that act as “*cis*-acting receptors” (66–68).

During initial MeV genome differentiation in the frontal cortex of this SSPE patient, the M-W125* truncation and the F-L454M mutation, detected in almost every MeV genome, may have enhanced the efficiency of the CADM-dependent membrane fusion process (37). Since W125* deletes the M protein carboxyl-terminal domain, this protein cannot dimerize (69). Interestingly, the M protein of another neurotropic negative-strand RNA virus that remains almost exclusively cell-associated, Borna disease virus, has a single domain of 142 amino acids that forms tetramers (70). Thus, MeV M-W125* may have disabled dimerization-dependent particle release while leaving other M protein functions intact.

In the brain of this SSPE patient, the M-F50S mutation promoted MeV spread in the context of the M-W125* truncation and of mutations of the F and H proteins (37). We show here that M-F50S drove MeV spread in hiPSC-derived neurons and astrocytes in the absence of any other mutation. This demonstration validates the inference that M-F50S drove neuropathogenesis in this SSPE case. Since in other SSPE cases mutations of the same amino acid, M-F50P and M-F50L, were identified (25, 26, 71), it is possible that these amino acid changes had similar impact on MeV spread in the brain of other SSPE patients.

But how did M-F50S promote ubiquitous brain spread? To assemble the viral RNP with the glycoproteins, the MeV M protein interacts with cytoskeletal components and with membrane lipids (69, 72–75). Since another mutation of the same amino acid, F50P, alters the interaction of M with filamentous actin (76), we are testing the hypothesis that M-F50S may affect the intracellular transport of viral components, possibly promoting their localization to those sites where intercellular infection transfer occurs.

It was reported that the mutation F-L454W renders a recombinant MeV hyperfusogenic and neuropathogenic, enhancing spread in murine organotypic brain cultures and in hiPSC-derived brain organoids (50). The recombinant MeV generated here with another amino acid at the same position, *MeV F-L454M*, was more fusogenic but did not spread preferentially in our hiPSC-derived neurons or in primary mouse neurons. Since tryptophan is a bulkier amino acid than methionine, it may have a distinct effect on the F protein stability and function (51).

A limitation of our study is that it reduces the complexity of the MeV genome population driving disease: at patient death, two genome sub-populations were present at variable ratios in different specimens, possibly due to distinct local selective pressures on mutations distributed on either genome (37). In contrast, here, we compared the spread of genomes with single mutations present at nearly 100% frequencies. In the context of the CIU hypothesis, we are seeking to characterize specific interactions between candidate disease-driving mutations by generating mixtures of genomes with individual mutations and assessing how these mixtures re-equilibrate when spreading in different host cells. We will also assess whether a single genome with multiple mutations, if rescuable, may compete with a multi-genome mutant mixture. A similar approach has brought insights into collaborative and competitive interactions of different F protein ectodomain mutants (77).

Another limitation is that we characterized infections in neurons and astrocytes cultured in isolation. However, MeV spread in the human brain may involve infection transfer between these two and other cell types (62). This can be addressed by performing infection studies in human cerebral organoids derived from hiPSC, which can include different cell types (50, 78). Animal models can also be considered, but the selective environment of the human and rodent brain is significantly different.

In conclusion, we showed that M-F50S promotes MeV spread in hiPSC-derived neurons and astrocytes, and in primary mouse neurons, in the absence of other mutations. This demonstration validates the inference that in this SSPE case M-F50S drove outward migration of an MeV genome population from the initial amplification site. Since subsequent brain spread depends on two complementary MeV genome subpopulations, we will seek to experimentally reproduce this infection phase through a genome population-based approach.

## MATERIALS AND METHODS

### Immunohistochemistry

Tissue samples were obtained from the brain of an SSPE patient as described previously (37). Tissue sections were cut at 5 µm thickness. Hematoxylin and eosin, CD45 (Dako, M0701), and CD11c (Leica, CD11C-563-L-CE) stainings were performed using Leica autostainer.

### Immunofluorescence and *in situ* hybridization

To deparaffinize and hydrate the formaldehyde fixed and paraffin-embedded (FFPE) tissue sections, slides were serially incubated for 10 min at room temperature in Xylene, 100% ethanol, 90% ethanol, 70% ethanol, and finally in water. For antigen retrieval, sections were steamed for 30 min in 1 mM EDTA. Sections were cooled, washed with water and phosphate-buffered saline (PBS) (SH30028.02, Cytiva) and then treated with TruBlack (23007, Biotium) for 15 min, washed with PBS, incubated with 0.25% Triton-X (Sigma-Aldrich), washed again with PBS, and blocked with 3% BSA (A7906, Sigma-Aldrich) for an hour at room temperature.

Primary antibodies included anti-MeV N_12_, raised against peptide containing nucleocapsid amino acids 12–33 NH_2_-KRNKDKPPITSGSGGAIRGIKH(C)-COOH (1:100); anti-MeV nucleocapsid mouse monoclonal (83KII, Millipore); Alexa Fluor 647 anti-NeuN (EPR12763, abcam); and anti-GFAP (Z0334, Dako). These antibodies were diluted in 1% BSA at a concentration of 1:100, 1:100, 1:100, and 1:50, respectively, and sections were incubated at 4°C overnight. Next day, sections were washed thrice with phosphate-buffered saline (PBS) and incubated with secondary antibody Alexa Fluor 488 or 647 at a concentration of 1:1,000 for 1 h. Sections were washed again with PBS and mounted with flouroshield media containing DAPI (f6057, Sigma-Aldrich). *In situ* hybridization was performed using smFISH as described previously (37).

Confocal microscopy for immunofluorescence and *in situ* hybridization was carried out using an LSM 980, AxioObserver Z1/7 microscope. Images were collected using a GaAsP PMT detector with 353, 548, 590, and 650 nm excitation lasers and a C-apochromat 40×/1.20 W Korr objective. Image processing and analysis were carried out using Zeiss ZEN Lite (Blue edition) version 3.5.

For quantification of MeV-positive neurons and astrocytes, raw TIFF images for each channel were exported and analyzed in ImageJ as described previously (37). Signal intensity from negative cells (cells negative for cell type and virus staining) was also determined and subtracted from intensity of positive cells to correct for background noise in each channel.

### Cell lines

293-4-46 cells (79) were maintained in DMEM supplemented with 10% fetal bovine serum (FBS, A5256701, Gibco) and 1.2 mg/mL of Geneticin G418 (10131035, Gibco). Vero-hSLAM cells (80) were maintained in DMEM supplemented with 10% FBS and 0.5 mg/mL of G418. HeLa-hSLAM cells (81) were grown in DMEM supplemented with 10% FBS and 0.1 mg/mL Zeocin (Gibco). The human lung cell line H358 (CRL-5807, ATCC) was maintained in RPMI 1640 medium supplemented with 2 mM L-glutamine and adjusted to contain a final concentration of 1.5 g/L sodium bicarbonate, 4.5  g/L glucose, 10  mM HEPES pH 7.2–7.5, 1  mM sodium pyruvate, nonessential amino acids, and 10% FBS.

### Mutagenesis

Site-directed mutagenesis was based on the Quick-Change system (200523, Agilent). It was performed in pCG-PmeIMV323-PmeIshort, which was generated in two steps. First, we transferred a NruI-FspI fragment from pMV323 (82) into pCG (83) and generated pCG-PmeIMV323-PmeI. Second, to reduce the size of the plasmid and allow efficient mutagenesis, we removed the region between PacI and AgeI, filled the ends with Klenow enzyme and dNTPs, and circularized the vector to produce pCG-PmeIMV323-PmeIshort.

To introduce the M-F50S mutation, pCG-PmeIMV323-PmeIshort was mutated using forward primer 5′ GGT GAT AGG AAG GAT GAA TGC T**C**T ATG TAC ATG TTT CTG CTG GG 3′ and reverse primer 5′ CC CAG CAG AAA CAT GTA CAT A**G**A GCA TTC ATC CTT CCT ATC ACC 3′ to generate pCG-PmeIMV323-PmeIshort M-F50S.

To introduce the F-L454M mutation, pCG-F wt (84) was mutated using forward primer 5′ CCT CCC ATA TCA **A**TG GAG AGG TTG GAC 3′ and reverse primer 5′ GTC CAA CCT CTC CA**T** TGA TAT GGG AGG 3′ to generate pCG-F-L454M.

The H-I8T mutation was introduced in two steps. First, shuttle plasmid pCG-(mCherryNLS)H was generated by removing a PacI-SpeI fragment coding for (mCherryNLS)H from p(+)MV323(mCherryNLS)H (16) and ligating it into pCG-Hwt (85) using PacI and SpeI. The newly generated pCG-(mCherryNLS)H was mutated to generate pCG-(mCherryNLS) H-I8T using forward primer 5′ A CAA CGA GAC CGA A**C**A AAT GCC TTC TAC 3′ and reverse primer 5′ GTA GAA GGC ATT T**G**T TCG GTC TCG TTG T 3′.

### Plasmids coding for full-length MeV genomes

Plasmids coding for full-length MeV genomes were generated in the IC323 background (82). Full length p(+)MV323(mCherryNLS)H contains an additional transcription unit with mCherry fused to a triple repeat nuclear localization signal (NLS) downstream of H (16). p(+)MV323(mCherryNLS)H M-F50S was generated by transferring a SacII restriction fragment coding for part of the P and M genes from pCG-PmeIMV323-PmeIshort M-F50S into p(+)MV323(mCherryNLS)H. p(+)MV323(mCherryNLS)H F-L454M was generated by transferring a NruI-PacI restriction fragment from shuttle plasmid pCG-F-L454M into p(+)MV323(mCherryNLS)H. p(+)MV323(mCherryNLS) H-I8T was generated by transferring a PacI-SpeI restriction fragment from shuttle plasmid pCG-(mCherryNLS) H-I8T into p(+)MV323(mCherryNLS)H.

### Virus rescue, stock production, and growth kinetics

Recombinant viruses were rescued as described previously (79). Passage 0 (p0) and passage 1 (p1) stocks were generated in Vero hSLAM cells as described previously (16). Viral titers were determined using the 50% tissue culture infectious dose (TCID50) method (86).

For growth kinetic analyses of the stocks, HeLa-hSLAM cells were seeded in 6-well plates at a density of 2.5 × 10^5^ cells per well. Next day cells were infected with p1 stocks at a multiplicity of infection (MOI) of 0.05. Supernatant and cell lysates were collected every 12 h for up to 72 hpi. Supernatants were centrifuged to get rid of debris before freezing at −80°C. Cell lysates collected in 500 µL Opti-MEM were subjected to three freeze-thaw cycles. Supernatant and cell lysate titers were determined on Vero-hSLAM cells.

### Immunoblotting

HeLa-hSLAM were seeded in 6-well plates. Next day, cells were infected with p1 stocks at MOI 0.3. Wells were harvested at 24 hpi in cell lysis buffer (9803, Cell Signalling) supplemented with protease inhibitors (11836153001, Roche) and incubated at 4°C for 30 min. Cell lysates were centrifuged to remove debris and supernatants were stored at −80°C. Proteins in cell lysates were quantified using the Bio-Rad protein assay dye reagent (5000006, Bio-Rad) based on the manufacturer’s protocol. 4%–15% Criterion Tris-HCl 4-precast protein gels (3450028, Bio-Rad) were used to run SDS-PAGE for all samples. Proteins were transferred to 0.45 µm PVDF transfer membrane (88518, Thermo Scientific) in Tris-Glycine transfer buffer (1610734, Bio-Rad) with 20% methanol using 330 mA current for 90 min. Membranes were blocked with 5% blotting-grade blocker (1706404, Bio-Rad) in Tris-buffered saline (TBS) for an hour and then incubated with primary antibodies overnight in 2.5% blotting-grade blocker (1706404, Bio-Rad). Primary antibodies against the MeV proteins were N_505_ (1:5,000) (87), M_81_ (46), and Fcyt (1:1,000) (29), as described previously. Antibody against the H ectodomain was raised against a peptide corresponding to amino acids 606-617 NH_2_-CTVTREDGTNRR-COOH (1:1,000). Secondary goat anti-rabbit antibody was used at a dilution of 1:10,000 and incubated for 1 h. For actin, HRP conjugated beta actin (1:10,000, MA5-15739-HRP, Invitrogen) was used. Pierce ECL Western blotting substrate (PI32106, Fisher Scientific) was used to visualize HRP. Images were taken using iBright 1500.

### Deep sequencing of virus stocks

To identify variable alleles in the virus stocks to be used for infection of neuronal cells, Vero-hSLAM cells were seeded at 3 × 10^6^ density in 100 mm dishes. One day after seeding, the cells were infected with passage 1 virus stocks at MOI 1. At >95% syncytia, cells were harvested in TRIzol reagent (15596018, Invitrogen), and RNA was extracted as per the manufacturer’s protocol. RNA pellets were resuspended in diethyl pyrocarbonate treated water and stored at −80°C.

For deep sequencing, the concentration and integrity of the RNA was assessed on the Agilent Bioanalyzer DNA 100 chip. cDNA library prep was conducted using Illumina’s Stranded Total RNA Prep, Ligation with Ribo-Zero Plus (20040529, Illumina) according to the manufacturer’s protocol. The 50 × 2 paired end sequencing of each library was performed on an MiSeq Reagent Kit v2-300 cycles (MS-102-2002, Illumina) (88). The raw Fastq files were uploaded into the Galaxy web platform (89).

For downstream processing and analysis of the data, we used the public server (http://usegalaxy.org/). Briefly, Illumina adapter sequences were clipped and low-quality reads were filtered using FASTQ by trimming reads from the 3′ end that had quality scores below or equal 20 (90). We used BWA version 0.7.17.5 to process reads and align them to MV323(mCherryNLS)H genomic sequence. IdxStats version 2.0.4 from the SAMTools software package was run to determine read count distributions across the reference sequences (91). The consensus genome aligned .bam files were then loaded in Integrative Genomics Viewer 2.3.98 and aligned reads were visualized (92, 93). Read count tables were generated using IGVTools. Allelic frequencies were calculated, and additional analyses were performed by uploading allelic frequencies into Microsoft Excel (16).

### Virus stock variable alleles

We noticed sequence variability of a few positions in different virus stocks, but variable nucleotides never exceeded 20% at any position in coding regions. Alleles occurring at 10%–20% frequency were as follows: in *MeV M-F50S,* U > C at position 6233 resulting in F-I259T at 17% frequency; in *MeV F-L454M,* C > U at position 2204 resulting in P/V-S133L and C-Q126* at 19% frequency (the same mutation resulted in changes in both the P/V and C overlapping reading frames); and in *MeV H-I8T,* C > A at position 3623 resulting in M-S62R at 16% frequency (GenBank: OR453544). Sequencing identified additional variable positions at >10% frequency in non-coding regions, particularly the long and GC-rich M-F intergenic region. However, since only a low number of reads spanning these regions were available, these observations may not be significant.

### Kinetics of recombinant MeV spread in receptor-positive cell lines

HeLa-hSLAM and H358 cells were seeded at a density of 2.5 × 10^5^ cells per well in 6-well plates. Next day, 3 wells per virus were infected at an MOI of 0.05 for 2 h. Fluorescent images were taken every 12 h up to 84 hpi. Fluorescent intensity was measured using ImageJ.

### Differentiation of iPSC-derived neurons and astrocytes and kinetics of MeV spread

HiPSC were differentiated into neurons or astrocytes as described previously (94). Neuronal progenitor cells were seeded at a density of 2 × 10^4^ cells/cm^2^ and differentiated into neurons for 2 weeks before infection. Differentiated astrocytes were cultured for 30 days for maturation and were seeded at a density of 2 × 10^4^ cells/cm^2^ the day before infection. On the day of infection, 3 wells were inoculated at an MOI of 0.5 for 2 h. Post-infection, images were taken every 5 days using the BZ-X810 microscope (Keyence) at 20× magnification. Fluorescent intensity was calculated using ImageJ.

### Culturing of embryonic mouse neurons in compartmentalized chambers

Embryonic mouse superior cervical ganglia (SCG) were dissected from E14 embryos (C57Bl/6J). SCG dissociation and compartmentalized culturing were performed as previously described (95). Briefly, isolated SCGs were washed with calcium-/magnesium-free Hank’s balanced saline solution (HBSS) and resuspended in 0.25 mg/mL trypsin (32P13847, Worthington Biochemical) in calcium-/magnesium-free HBSS for dissociation and incubated for 15 min in a 37°C water bath. Trypsinized SCGs were centrifuged and resuspended in 1 mg/mL trypsin inhibitor (9035-81-8, Gibco) in calcium-/magnesium-free HBSS and then incubated for 5 min in a 37°C-water bath. Next, SCGs were centrifuged and resuspended in complete neurobasal (21103049, Invitrogen, Neurobasal), 1 × pen/strep/Glu (15140-22, Invitrogen), 1x B-27 (17504-044, Gibco), and 60 ng/mL 2.5S NGF (820202, Invitrogen) and dissociated by trituration using a 5 mL Pasteur pipette. After dissociation, neurons were seeded into one lateral compartment of the Campenot chambers previously coated with poly-DL ornithine (P8638, Millipore-Sigma) and murine laminin (23017, ThermoFisher) at approximately 1 SCG per chamber. Forty-eight hours post seeding, neuronal media was aspirated and substituted with fresh complete neurobasal supplemented with 1 µM Cytosine-β-d arabinofuranoside (C6645, Sigma-Aldrich) for 24 h to eliminate mitotically active cells, leaving only viable neurons in the culture. Neuron cultures were fed with fresh complete neurobasal every 3–4 days. Neurons were cultured for 17 days to allow maturation and neurite growth prior to MeV infection.

### Infection of primary mouse neuron cultures

Compartmentalized SCG neuron cultures were overlaid with MeV-infected H358 cells as previously described (53). H358 cells were seeded into 24 well plates and maintained for 2 days prior to inoculation with MeV at 0.5 PFU/cell. One hour after inoculation, infected H358 cells were washed with Dulbecco’s phosphate-buffered saline (DPBS) and given fresh H358 media. Four hours after inoculation, H358 cells were trypsinized and resuspended in complete neurobasal +5% FBS (vol/vol) and subsequently seeded onto SCG axons in the middle compartment at 2 x 10^4^ MeV-infected H358 cells per compartment. Throughout SCG neuron infection, lateral compartments were given fresh complete neurobasal every 3 days, and middle compartments containing MeV-infected H358 cells were given fresh complete neurobasal with 5% FBS (vol/vol) every 3 days.

### Live-cell quantification and imaging of MeV-infected mouse neuron cultures

Live-cell epifluorescence imaging of SCG neurons was performed on a Nikon Ti-Eclipse inverted microscope (Nikon Instruments) equipped with a Spectra X LED excitation module (Lumencor) and fast-switching emission filter wheels (Prior Scientific). Fluorescence imaging used paired excitation/emission filters and dichroic mirrors for Tetramethylrhodamine (TRITC) filters (Chroma Corp.). All brightfield images were acquired using phase-contrast configuration at 10× optical magnification.

Imaging was performed on the microscope in conjunction with a stage-top incubation system (Quorum Scientific). Infected SCG neuron cultures were maintained at 37°C in a 5% (vol/vol) CO_2_-enriched atmosphere using a stage-top incubator system. TRITC channel fluorescent illumination intensity was set to 50% power at 100 ms exposure time to avoid photobleaching and phototoxicity. At the times indicated, MeV infection of SCG neurons was quantified by counting the number of mCherry+ soma in the lateral compartment that dissociated SCGs were seeded.

### Sequencing analysis of *MeV M-F50S* after replication in primary mouse neurons

Mouse SCG neurons infected with *MeV M-F50S* were pooled from four separate chambers and harvested in Trizol (Invitrogen). RNA was extracted using the manufacturer’s protocol. Library preparation, Illumina sequencing, and downstream analysis were performed as described above for deep sequencing of virus stocks.

## Data Availability

All data critical to this study are presented in the paper. Additional data are available from the corresponding author on request.
